# Comparative metabolomic analysis in plasma and cerebrospinal fluid of humans and in plasma and brain of mice following antidepressant-dose ketamine administration

**DOI:** 10.1038/s41398-022-01941-x

**Published:** 2022-05-02

**Authors:** Ruin Moaddel, Panos Zanos, Cristan A. Farmer, Bashkim Kadriu, Patrick J. Morris, Jacqueline Lovett, Elia E. Acevedo-Diaz, Grace W. Cavanaugh, Peixiong Yuan, Mani Yavi, Craig J. Thomas, Lawrence T. Park, Luigi Ferrucci, Todd D. Gould, Carlos A. Zarate

**Affiliations:** 1 Biomedical Research Center, National Institute on Aging, National Institutes of Health, Baltimore, 21224 MD USA; 2 Departments of Psychiatry, Pharmacology, and Anatomy and Neurobiology, University of Maryland School of Medicine, Baltimore, 21201 MD USA; 3 Department of Psychology, University of Cyprus, 2109 Nicosia, Cyprus; 4 Experimental Therapeutics and Pathophysiology Branch, National Institute of Mental Health, National Institutes of Health, Bethesda, MD USA; 5 Division of Preclinical Innovation, National Center for Advancing Translational Sciences, National Institutes of Health, Rockville, MD 20850 USA; 6 Veterans Affairs Maryland Health Care System, Baltimore, MD 21201 USA

**Keywords:** Predictive markers, Clinical pharmacology

## Abstract

Subanesthetic-dose racemic (*R,S*)-ketamine (ketamine) produces rapid, robust, and sustained antidepressant effects in major depressive disorder (MDD) and bipolar disorder (BD) and has also been shown to effectively treat neuropathic pain, complex regional pain syndrome, and post-traumatic stress disorder (PTSD). However, to date, its mechanism of action remains unclear. Preclinical studies found that (2 *R*,6 *R*;2 *S*,6 *S*)-hydroxynorketamine (HNK), a major circulating metabolite of ketamine, elicits antidepressant effects similar to those of ketamine. To help determine how (2 *R*,6 *R*)-HNK contributes to ketamine’s mechanism of action, an exploratory, targeted, metabolomic analysis was carried out on plasma and CSF of nine healthy volunteers receiving a 40-minute ketamine infusion (0.5 mg/kg). A parallel targeted metabolomic analysis in plasma, hippocampus, and hypothalamus was carried out in mice receiving either 10 mg/kg of ketamine, 10 mg/kg of (2 *R*,6 *R*)-HNK, or saline. Ketamine and (2 *R*,6 *R*)-HNK both affected multiple pathways associated with inflammatory conditions. In addition, several changes were unique to either the healthy human volunteers and/or the mouse arm of the study, indicating that different pathways may be differentially involved in ketamine’s effects in mice and humans. Mechanisms of action found to consistently underlie the effects of ketamine and/or (2 *R*,6 *R*)-HNK across both the human metabolome in plasma and CSF and the mouse arm of the study included LAT1, IDO1, NAD^+^, the nitric oxide (NO) signaling pathway, and sphingolipid rheostat.

## Introduction

Racemic (*R,S*)-ketamine (ketamine) has been in use since 1970 as a rapid-acting anesthetic [1] and has been listed on the WHO’s essential medicine list since 1985. Recent studies found that a single, subanesthetic-dose ketamine infusion produced rapid and sustained antidepressant effects in individuals with major depressive disorder (MDD) [2, 3] and bipolar depression (BD) [4]. Ketamine also appears to effectively treat neuropathic pain [5], complex regional pain syndrome [6, 7], pre-and post-operative pain [8], and post-traumatic stress disorder (PTSD) [9, 10], in addition to having analgesic [11] and anti-inflammatory effects [8, 12].

Despite this promising treatment profile, subanesthetic-dose ketamine has adverse effects including dissociation, cognitive impairment, and psychotomimetic effects, as well as significant abuse liability [13–15]. These adverse effects have been attributed to *N*-methyl-D-aspartate receptor (NMDAR) inhibition, a mechanism of action proposed to underlie ketamine’s antidepressant effects [16]. Nevertheless, several other NMDAR antagonists do not elicit a similar antidepressant pharmacological response [17], suggesting that other mechanisms underlie ketamine’s rapid antidepressant properties [15]. As a result, ketamine’s precise antidepressant/analgesic mechanism of action remains largely unknown. Other proposed mechanisms include: blockage of the hyperpolarization-activated cyclic nucleotide gated potassium channel 1 (HCN1) [18]; involvement of the cholinergic, aminergic, and opioidergic systems [18]; increased mitochondrial activity, synaptogenesis and mammalian target of rapamycin (mTOR) signaling pathways; inhibitors of group II mGlu receptors [19]; and protein synthesis via eukaryotic elongation factor-2 (eEF2) kinase inhibition [20, 21].

In this context, one alternative mechanism that may underlie ketamine’s antidepressant effects is related to its metabolites. Ketamine is extensively metabolized to a large number of downstream metabolites including norketamine, dehydronorketamine, hydroxyketamines, and hydroxynorketamines (HNKs) [4, 7, 15]. In particular, (2 *S*,6 *S*;2 *R*,6 *R*)-HNK was found post-ketamine administration in the plasma of human participants [4, 7, 22] as well as in the brain and plasma of mice [23]. In rodent models, (2 *R*,6 *R*)-HNK was subsequently found to elicit antidepressant-like effects similar to those of ketamine but without its adverse effects [21, 23–31]. (2 *R*,6 *R*)-HNK was also found to induce analgesic effects in preclinical models of pain [32]. In terms of potential mechanisms of action, both ketamine and (2 *R*,6 *R*)-HNK upregulated α-amino-3-hydroxy-5-methyl-4-isoxazolepropionic acid (AMPA) receptors in vivo [23] but, when compared directly, only (2 *R*,6 *R*)-HNK upregulated AMPA receptors in vitro [33]. Deciphering the exact mechanism of both ketamine’s and (2 *R*,6 *R*)-HNK’s antidepressant activity remains a major priority in the field.

Metabolomics provides a comprehensive analysis of lipids, amino acids, biogenic amines, and other metabolic products within a given biological subject [34]. Thus, determining metabolite changes in cerebrospinal fluid (CSF) post-ketamine administration may capture a metabolic signal that is closer to ketamine’s mechanism of action, thereby helping identify its effects on the central nervous system (CNS). To date, most metabolomic studies with ketamine have been limited to using plasma from humans and/or rodents [35–37]. This exploratory study was a targeted metabolomic analysis carried out on the plasma and CSF of healthy human participants who received a 40 min intravenous infusion of ketamine (0.5 mg/kg) to determine whether changes in the plasma corresponded to similar changes in the CSF. Several specific metabolomes were also targeted that have been shown to play a role in depression and/or neurodegenerative diseases, including the kynurenine (KYN) [35] and nicotinamide adenine dinucleotide (NAD^+^) metabolomes [38–40]. To help determine the contribution of (2 *R*,6 *R*)-HNK to changes in the metabolomic profile observed following ketamine treatment, a parallel targeted metabolomic analysis was carried out in mice that received either 10 mg/kg ketamine, 10 mg/kg of (2 *R*,6 *R*)-HNK, or saline; the mouse metabolomic analysis was carried out in plasma, hippocampus, and hypothalamus.

## Materials and methods

### Human participants

Nine healthy human volunteers aged 19–36 years (mean age = 27 ± 6) (Supplementary Table S1) participated in the study. Healthy control subjects consisted of males and females, with no Axis I disorder as determined by SCID-NP. Healthy control subjects were free of medications affecting neuronal function or cerebral blood flow or metabolism. Subjects in both groups were in good physical health as determined by medical history, physical exam, blood labs, electrocardiogram, chest x-ray, urinalysis, and toxicology.

Healthy volunteers received a ketamine infusion (0.5 mg/kg/40 min IV) (Mylan Institutional, Galway, Ireland) and contributed plasma and CSF samples for up to 28 h. Blood and CSF were collected at baseline, 40 min, 120 min, and 230 min, as well as at 6, 10, 12, 22, 24, 26, and 28 h post-infusion. Parallel blood draws with a 48 h timepoint were also collected for blood. Whole blood samples were collected using BD vacutainer tubes with heparin and centrifuged at 3000 rpm at 4 °C for 10 min; separated plasma samples were aliquoted and stored at −80 °C until assay. All participants provided written consent prior to study entry, and this study was approved by the Combined Neuroscience Institutional Review Board of the NIH (NCT03065335).

### Mice

Male CD-1 mice nine weeks of age received a single intraperitoneal (i.p.) (10 ml/kg) injection of saline, 10 mg/kg of ketamine hydrochloride, or 10 mg/kg of (2 *R*,6 *R*)-HNK hydrochloride. Samples were collected at baseline (no injection) (*n* = 8) and at four timepoints post-administration (15 min, 60 min, 240 min, and 24 h) in different cohorts of mice. These doses of ketamine and (2 *R*,6 *R*)-HNK were based on doses previously found to effectively induce antidepressant-like effects in the same mouse strain [23, 26]. For tissue collection, mice were exposed to 3.5% isoflurane for two minutes and then immediately decapitated. Trunk blood was collected in EDTA-containing Eppendorf tubes, on ice. Concomitantly, the hippocampus and hypothalamus were excised using a mouse-brain matrix on ice. 100 µl of whole blood was also collected in a separate tube. All samples were frozen on dry ice immediately after collection. Eight samples were collected for each timepoint and treatment. All experimental procedures were approved by the University of Maryland Baltimore Animal Care and Use Committee and were conducted in full accordance with the National Institutes of Health Guide for the Care and Use of Laboratory Animals.

### Metabolomics panels

#### Metabolomic assay

A targeted metabolomic assessment was carried out following the manufacturer’s protocol using Biocrates’ MxP® Quant 500 kit (Biocrates, Innsbruck, Austria), which quantifies 630 distinct metabolites in human and rodent plasma (10 µl), human CSF (10 µl), and mouse brain (10 µl). For the mouse hypothalamus and hippocampus, tissue was homogenized following a previously published protocol [41] with slight modifications. Additional details can be found in the Supplementary Materials.

#### Kynurenine metabolome

Separation of the kynurenines (KYNs) was accomplished as previously described [35, 42]. Relative concentrations of the metabolites were determined in standard solution using area ratios calculated using a deuterated standard; because matrix effects were not considered, these only provide a measure of relative abundance and not absolute quantification. All data were normalized to a pooled sample of study participants that was run each day.

#### NAD^+^ metabolome

Separation of the NAD^+^ metabolites was accomplished as previously described [38, 39]; additional details can be found in the Supplementary Materials. Briefly, relative values for the metabolites were determined using area ratios of the targeted metabolites, and the corresponding internal standard and data were normalized to the pooled samples.

### Statistical analysis

Because this was an exploratory analysis, no a priori power calculations were performed. Following guidelines established by the American Statistical Association [43], uncorrected *p*-values for two-tailed tests focusing on the magnitude and variability (confidence intervals) in effects are reported. Test assumptions were confirmed via visual inspection. Parameter estimates, test statistics, and uncorrected *p*-values for all comparisons are provided in the Supplementary Materials.

#### Human data

Data were log transformed prior to analysis. Values were Pareto-scaled to baseline. A principal components analysis (PCA) was performed on the Pareto-scaled data to reduce the number of features in the data, using the prcomp package for R version 4.0.2. The number of components was selected using a combination of eigenvalues >1 and visual inspection of the scree plot. The effect of time (human) on the component scores was examined and, to account for repeated observations, a random subject intercept in a generalized linear model was used. The parameter estimates of interest were the estimated marginal means at each timepoint, given that Pareto scaling allows interpretation of these values as change from baseline. Based on the results of those analyses, individual analytes were selected and underwent univariate analysis, using the same model.

#### Mouse data

Data were log transformed prior to analysis. Area under the curve (AUC) for serial sacrifice design was calculated using the PK package for R version 4.0.2 [44]. The AUC of each metabolite was compared between groups using a Z-transformation. The NAD^+^ metabolome, comprising only a few features, was investigated using univariate analysis, wherein concentrations were Pareto-scaled to the control group and evaluated using a general linear model with fixed effects of time and group. The parameter estimates of interest were the between-group contrasts at each timepoint.

## Results

### Metabolomic analysis in healthy human volunteers

Of the 630 potential metabolites quantified in the targeted metabolomic analysis of plasma and CSF samples from nine human volunteers, metabolites that had quantitative values in less than 67% of the total samples were excluded, leaving 466 metabolites in the plasma and 82 in the CSF. Circulating NAD^+^ levels in the CSF were also determined. In the PCA carried out with the metabolomic dataset and based on the scree plot, four components in CSF and seven components in plasma were carried forward, which explained 57% and 76% of the variance, respectively. The metabolites with the strongest loadings for each of these components are shown in Supplementary Fig. S1 and Supplementary Table S2. Only one component for plasma and CSF, PC2, exhibited a consistent pattern of change over time post-ketamine, where it steadily increased followed by a decrease by 28 h in the CSF and 48 h in plasma (Supplementary Fig. S1).

### Plasma metabolomics

**Fig. 1:**
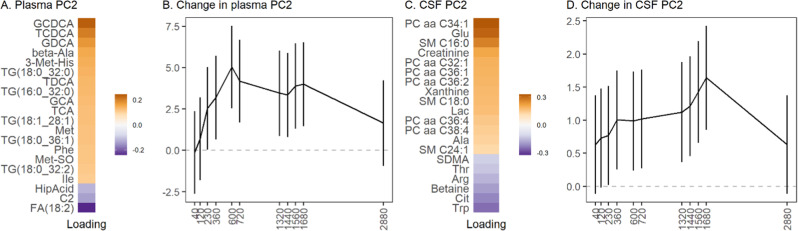
Selected results of principal components analysis (PCA) on human metabolome. **A**, **C** Features with the 20 strongest loadings on PC2 in plasma (**A**) and cerebrospinal fluid (CSF) (**C**). **B**, **D** Result of mixed model estimating mean (95% CI) baseline-normalized PC2 values at each timepoint in plasma (**B**) and CSF (**D**). Y-axis is a PC score and has no units.

In the plasma compartment, the major contributors to the PC2 were bile acids, leucine, isoleucine, glycine, phenylalanine, methionine, cystine, 3-Met-His, and betaine, all of which loaded positively with PC2 (Fig. 1, Supplementary Table S2A). In the univariate analysis (Supplementary Table S3), all seven identified bile acids followed a similar pattern, with a steady increase at 40 min post-infusion that peaked at six hours, then decreased until 12 h, and returned to baseline at 48 h. The amino acids followed the same general pattern, with circulating levels near baseline for between two to four hours post-infusion, followed by increasing circulating levels peaking at 10 h (phenylalanine and methionine) or 12 h (leucine and isoleucine), then remaining steady before decreasing; levels had not returned to baseline at 48 h post-infusion. Of the ratios calculated, only the KYN/tryptophan ratio decreased with ketamine treatment. Branch chain amino acid (BCAA) (sum of leucine, isoleucine, and valine) levels increased following ketamine treatment, and the arginine/asymmetric dimethylarginine (ADMA) ratio trended higher following ketamine treatment. Acetylcarnitine (C2), identified as one of the major contributors to the PCA, decreased two hours post-infusion, remained steady for the rest of the study, and did not return to baseline after 48 h.

Of the lipids, lysophosphatidylcholine (LPC) a C18:2 and LPC a C20:4 were identified as major contributors to PC2 in plasma. Both increased post-ketamine, peaked near 10 h, and remained steady until 48 h. A negative trend for fatty acid (FA) 18:2 was observed post-ketamine, with a maximal decrease four hours post-infusion that did not return to baseline for 48 h. SM C18:1 also had a decreasing trend post-ketamine. Increasing levels of several diacylglycerols (DGs) and triglycerides (TGs) containing FA 18:2 were also observed (Supplementary Table S3). Several TGs were found to be major contributors to PC2 (Fig. 1; Supplementary Tables S2 and S3).

### CSF of human healthy controls

In the CSF, amino acids, amino acid-related metabolites, sphingomyelins, and phosphatidylcholines were the major contributors that changed post-infusion (Fig. 1; Supplementary Tables S2B and S4). The bile acids measured in the CSF were either below or at the lower limit of quantitation. No LPC was detectable in more than 50% of the samples in the CSF, and no TGs were detectable in the CSF. NAD^+^ levels decreased subtly in the CSF post-ketamine, then steadily decreased with a maximal drop at 22 h, finally returning near baseline at 26 h. In the univariate analysis (Supplementary Table S4), creatinine steadily increased post-infusion, and SM C16:0, SM C18:0, and SM C18:1 trended positively post-ketamine, peaking near 12 h post-infusion, remaining steady, and not returning to baseline 28 h post-infusion. PCaa C32:1, PCaa C34:1, and PCaa C36:1 also increased, peaking at 24 h. Tryptophan decreased post-ketamine in a biphasic manner, with a steady decrease until six hours, a moderate recovery returning to a maximal decrease at 24 and 26 h, and finally returning to baseline at 28 hours. Symmetric dimethylarginine (SDMA), an endogenous inhibitor of nitric oxide synthase (NOS), had a negative association with ketamine treatment in the CSF, and the arginine/SDMA ratio, which reflects nitric oxide (NO) production, increased post-ketamine. However, the global arginine bioavailability ratio (GABR), a measure that estimates NO synthetic capacity in vivo [35], was not associated with ketamine treatment. Of the remaining ratios, BCAAs decreased post-ketamine. The putrescine/ornithine ratio initially increased, then decreased six hours post-infusion, and did not return to baseline until 28 h post-infusion. Interestingly, several metabolites loaded in opposite directions onto the plasma and CSF components post-ketamine, including leucine [0.09/−0.09], isoleucine [0.11/−0.09], tryptophan [0.02/−0.25], methionine [0.12/−0.10], and tyrosine [0.07/−0.07], which loaded positively on plasma and negatively on CSF, respectively, suggesting decreased transport into the CSF post-ketamine.

### Metabolomic analysis in mice

**Fig. 2:**
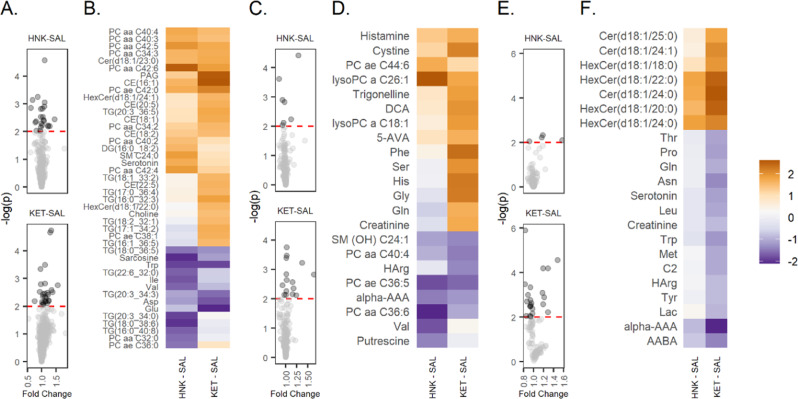
Mouse AUC comparisons. The area under the curve (AUC) of each metabolite was compared using Z-transformation between groups receiving 10 mg/kg ketamine (KET), 10 mg/kg (2 *R*,6 *R*)-hydroxynorketamine (HNK), or saline (SAL). Volcano plots show the p-value for the between-group comparison against fold change in feature. Heatmaps show the test statistics (Z) for features with at least one -log(p) > 2. All heatmaps share the legend at far right. **A** Volcano plot for plasma MxP500. **B** Heatmap for plasma MxP500. **C** Volcano plot for hypothalamus features. **D** Heatmap for hypothalamus features. **E** Volcano plot for hippocampus features. **E** Heatmap for hippocampus features.

Of the 630 metabolites investigated with the MxP 500 platform, 337 were above the limit of detection in >67% of the mouse plasma samples available for data analysis. Similar results were found for 185 metabolites in the hypothalamus and 96 metabolites in the hippocampus. The AUC calculated for serial sacrificed design of each metabolite was compared using a Z-transformation between groups receiving 10 mg/kg ketamine, 10 mg/kg (2 *R*,6 *R*)-HNK, and saline in mouse plasma (Supplementary Table S5), mouse hippocampus (Supplementary Table S6), and hypothalamus (Supplementary Table S7). Test statistics (Z) for the group comparison are plotted for features with at least one -log(p) > 2 (Fig. 2).

### Plasma metabolomics

The results of the univariate metabolomic analysis in mice revealed that four metabolites changed following treatment with either ketamine or (2 *R*,6 *R*)-HNK, with tryptophan decreasing following treatment and three PCs (PC aa C42:5, PC aa C42:6, PC ae C42:0) increasing following treatment (Fig. 2; Supplementary Table S5). From the NAD^+^ metabolites (Supplementary Table S8), only NADP^+^ and NADPH changed in both ketamine and (2 *R*,6 *R*)-HNK treated mice, with NADP^+^ decreasing at 15 min post-treatment and NADPH decreasing at 15 and 60 min post-treatment.

In (2 *R*,6 *R*)-HNK treated mice, serotonin, three PCs (PC aa C40:3, PC aa C40:4, PC aa C42:4) and SM C24:0 increased following treatment, with sarcosine, valine, isoleucine, and four TGs (TG(16:0_40:8), TG (18:0_38:6), TG(20:3_34:0), TG(22:6_32:0)) decreasing post-treatment (Fig. 2; Supplementary Table S5). From the NAD^+^ metabolites (Supplementary Table S8), only NAD^+^ decreased slightly at 15 min post- (2 *R*,6 *R*)-HNK treatment. The nicotinamide mononucleotide (NMN): nicotinamide (NAM) ratio, an indication of nicotinamide phosphoribosyltransferase (NAMPT) activity, decreased at 15 minutes in (2 *R*,6 *R*)-HNK-treated mice.

In ketamine-treated mice, glutamate and aspartate decreased post-treatment (Fig. 2, Supplementary Table S5), and PAG, PC ae C38:1, PC aa C34:3, three TGs (TG (16:0_32:3) TG(17:1_34:2), TG (20:3_36:5)), ceramide (Cer) (d18:1/23.0), and cholesterol ester (CE) 18:1, CE 20:5, and CE 16:1 increased post-ketamine. For the NAD^+^ metabolites, NAM increased slightly at 24 h post-ketamine (Supplementary Table S8). Although, no ratios were found to change following ketamine treatment, at 240 min NAD^+^ kinase (NADP/NAD^+^) was higher in (2 *R*,6 *R*)-HNK-treated mice relative to ketamine-treated mice, and NMNAT (NAD^+^/NMN) were higher in ketamine-treated mice relative to (2 *R*,6 *R*)-HNK-treated mice.

### Hippocampus and hypothalamus

Univariate analyses found that only Hex Cer (d18:1/24:0) increased in the hippocampus (-log(p) > 2) following both ketamine and (2 *R*,6 *R*)-HNK treatment. No other metabolites were found to change following (2 *R*,6 *R*)-HNK treatment in the hippocampus. However, several additional metabolites decreased following ketamine treatment, including asparagine, α-Amino adipidic acid (α-AAA), α-aminobutyric acid (AABA), tryptophan, proline, serotonin, threonine, leucine, methionine, glutamine and C2 (Fig. 2; Supplementary Table S6); in addition, several ceramides (Cer(d18:1/24:1), Cer (d18:1/24:0), HexCer (d18:1/18:0), HexCer (d18:1/20:0), Hex Cer (d18:1/22:0)) increased post-ketamine.

In the hypothalamus, univariate analysis found that LPC a C26:1 increased and PC ae C36:5 decreased in both ketamine and (2 *R*,6 *R*)-HNK treated mice (Fig. 2; Supplementary Table S7). Two additional metabolites decreased following (2 *R*,6 *R*)-HNK treatment: valine and PC aa C36:6. In ketamine-treated mice, phenylalanine, histidine, glycine, cystine, deoxycholic acid (DCA), serine, trigonelline, and LPC a C18:1 increased post-treatment.

## Discussion

**Fig. 3:**
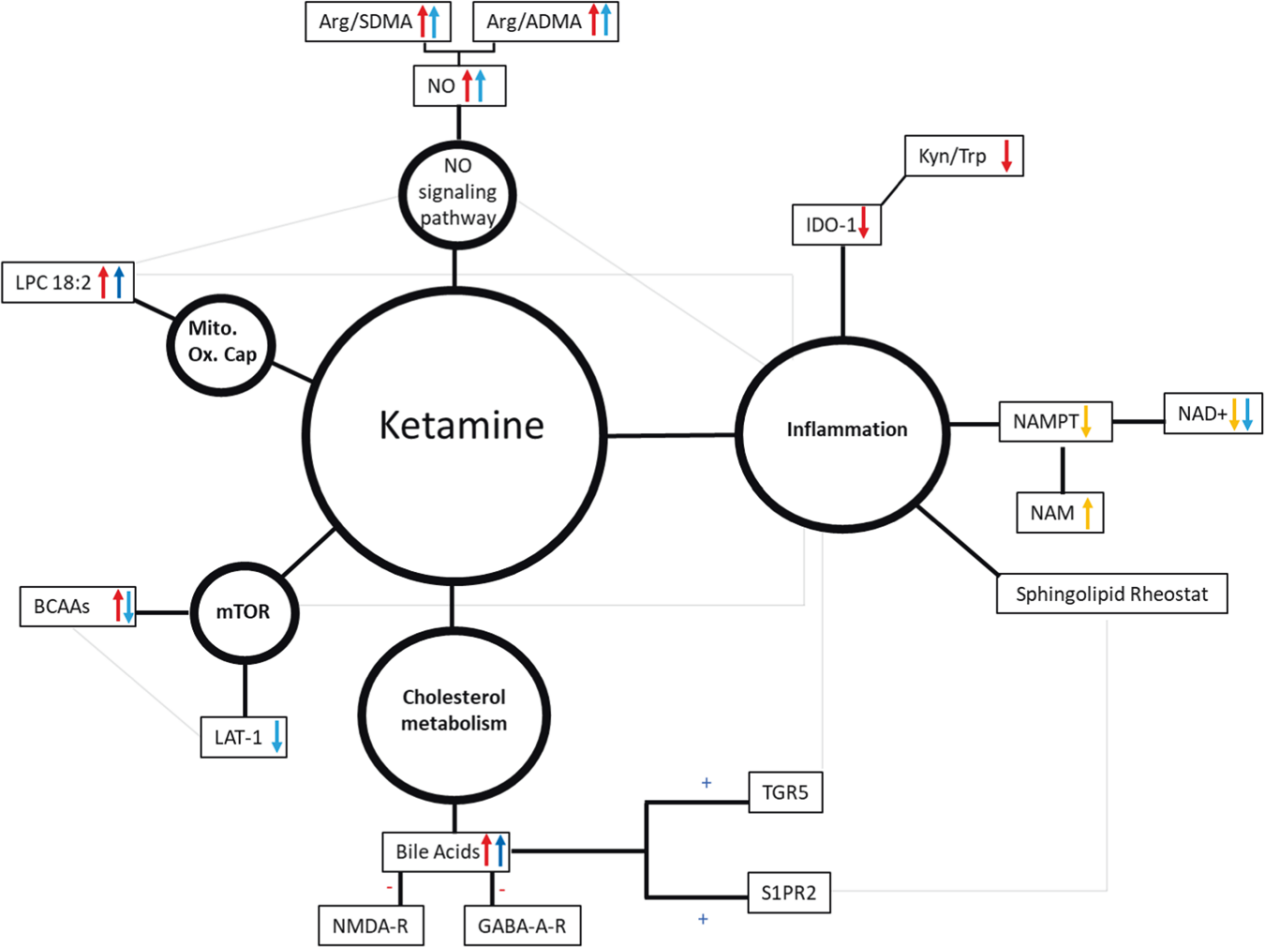
Potential mechanism identified for ketamine and/or (2 *R*, 6 *R*)-hydroxynorketamine (HNK). Overview of the exploratory metabolomic results on the potential mechanism of ketamine and/or (2 *R*,6 *R*)-HNK, including inflammation, the nitric oxide (NO) signaling pathway, cholesterol metabolism, mammalian target of rapamycin (mTOR) and/or mitochondrial oxidative capacity. Directional changes of the metabolites and/or ratios are indicated as well as the source (red: human plasma; light blue: cerebrospinal fluid (CSF); yellow: mouse plasma and/or whole blood; dark blue: mouse brain). Light grey lines indicate additional interactions.

This longitudinal study used metabolomics to explore the putative mechanisms underlying ketamine’s therapeutic effects while also assessing the relative metabolomic differences between (2 *R*,6 *R*)-HNK and ketamine to provide insights into their respective mechanisms of action. The targeted metabolomic study was carried out in the plasma and CSF of nine healthy human volunteers who received a 40 min ketamine infusion (0.5 mg/kg), and a parallel targeted study was carried out in plasma, hippocampus, and hypothalamus of mice receiving either 10 mg/kg of ketamine, 10 mg/kg of (2 *R*,6 *R*)-HNK, or saline. Ketamine and (2 *R*,6 *R*)-HNK metabolomic changes were shown to affect multiple pathways, several of which are associated with inflammation (Fig. 3).

Several studies have suggested that ketamine’s antidepressant effects are mediated via the mTOR signaling pathway [45, 46], and pre-clinical evidence also suggests that (2 *R*,6 *R*)-HNK exerts mTOR-dependent effects [21, 26, 29–31]. Ketamine-induced mTOR activation has been associated with a short-term decrease in depressive symptoms in MDD patients, and this activation may be due to increased circulating levels of BCAAs [47]. In this context, in the present study BCAAs increased in the plasma of healthy volunteers post-ketamine but decreased in the CSF, echoing similar findings in both the hippocampus and hypothalamus of ketamine- and (2 *R*,6 *R*)-HNK-treated mice, respectively. Whether the decrease in the CSF reflects a decrease in mTORC1 activation is unknown, given that circulating CSF metabolite levels reflect the extracellular environment and may not accurately reflect intracellular changes, which may increase as a result of intracellular compensatory mechanisms. Tryptophan, phenylalanine, and arginine were also found to decrease in CSF following ketamine treatment, with tryptophan also decreasing in the hippocampus of ketamine-treated mice. These amino acids are all substrates for the large neutral amino acid (LAT-1) (SLC7A5) transporter [48], suggesting that ketamine may induce decreased transport across the blood-brain barrier via LAT-1. This would be consistent with a recent study showing that leucine induced antidepressant-like effects by blocking KYN uptake via LAT-1 in a mouse model of LPS-induced depressive-like behaviors [49].

KYN is metabolized to several metabolites involved in the interface of inflammatory/immune response and glutamatergic neurotransmission [50]. IDO1, a therapeutic target in depression, metabolizes tryptophan to KYN and is upregulated during pro-inflammatory states induced by several cytokines (IL-1β, IL-6, IFN-α, TNF-α) [51, 52]. In this study, the KYN/tryptophan ratios, a marker of IDO activity, decreased in the plasma of healthy volunteers post-ketamine, consistent with ketamine’s anti-inflammatory effects. The KYN pathway is also the *de novo* synthetic pathway for NAD^+^, an important cofactor for several biochemical pathways [37, 53, 54]. The major pathway for generating NAD^+^ is the salvage pathway, with NAMPT as the rate-limiting enzyme. Surprisingly, in the present study, NAD^+^ levels decreased following treatment in both the CSF of healthy volunteers as well as the whole blood of (2 *R*,6 *R*)-HNK-treated mice. Although, increased NAD^+^ levels are believed to be neuroprotective [39, 55], under inflammatory conditions, NAD^+^ is preferentially released from intracellular stores [56], and intracellular NAMPT (iNAMPT) is secreted to the extracellular space (eNAMPT) [57, 58]. In vitro NAMPT inhibition has been shown to reduce pro-inflammatory cytokine secretion by inflammatory cells [58], and NAMPT inhibition via FK-866 improved inflammation-related disease in animal models [59]. In the mouse arm of the study, NAMPT activity decreased in (2 *R*,6 *R*)-HNK-treated mice, suggesting that (2 *R*,6 *R*)-HNK may act via NAMPT. NAM levels were also increased in the whole blood of ketamine-treated mice and showed a trend towards increasing in (2 *R*,6 *R*)-HNK-treated mice at several timepoints. Notably, increased NAM levels are thought to have beneficial anti-inflammatory [60] and antidepressant [60, 61] effects.

Although, the signaling molecule NO has also been thought to play a role in inflammation as well as in ketamine’s antidepressant effects [35, 62], a recent study suggested that the NO pathway may not play a primary role in these effects [63]. Multiple factors affect NO levels, including arginase activity, amino acid levels, NOS activity, and the presence of specific endogenous NOS inhibitors, such as ADMA and/or SDMA [34]. GABR, an estimate of NO synthetic capacity in vivo, was previously demonstrated to increase in plasma following ketamine treatment in MDD responders relative to non-responders [34], with no change in healthy volunteers. In this study, GABR did not change in the plasma or CSF of healthy volunteers post-ketamine. However, the arginine/SDMA ratio and the arginine/ADMA ratio, which reflects NO production, were both increased in human CSF and plasma. This is consistent with previous reports showing that the selective serotonin reuptake inhibitor (SSRI) paroxetine increases circulating NO levels in healthy volunteers [64] and suggests that ketamine may act through the NO cycle via the bioavailability of arginine.

Ketamine also increased levels of several bile acids in the plasma of healthy volunteers (Fig. 1), suggesting that cholesterol metabolism may play a role in this agent’s mechanism of action [65]. A recent study found that lower serum bile acid concentrations were associated with markers of dementia, suggesting that cholesterol catabolism and bile acid synthesis may impact the progression of dementia [66]. Several receptors are activated by the bile acids, including the nuclear receptors FXR, PXR, and vitamin D3 receptor, as well as membrane receptors such as Takeda G-protein-coupled receptor 5 (TGR5) and sphingosine-1-phosphate receptor 2 (S1PR2) [67, 68]. TGR5 activation induces key anti-inflammatory pathways [67] and, as a result, may play a role in ketamine’s antidepressant effects. NMDA and GABA_A_-R are also well-studied bile acid-regulated receptors in the brain, where they were shown to function as NMDAR and GABA_A_-R antagonists [67], and chenodeoxycholic acid (CDCA) and taurochenodeoxycholic acid (TCDCA) are the most effective at reducing the activation of these receptors [67]. CDCA and TCDCA also preferentially inhibit GluN2D and GluN3B containing NMDARs, followed by GluN2A- and GluN2B-containing NMDARs [69]. While high circulating cholesterol is a risk factor for dementia in young and middle-aged individuals, whether peripheral cholesterol levels directly impact brain function remains unknown [70]. However, considering that cholesterol homeostasis is carried out by the same protein network in the CNS as in the liver [71], and that ketamine and its metabolites cross the blood-brain barrier [23, 26, 29, 72], a potentially similar increase in the CSF could be expected. This is consistent with a recent study that implicated tyrosine kinase receptor 2 (TRKB) in the mechanism of antidepressant efficacy, including for ketamine [73]. Interestingly, TRKB signaling is bidirectionally linked to brain cholesterol metabolism [73]. In our study, none of the bile acids were found at quantitative levels in the CSF, but DCA increased post-ketamine in mouse hypothalamus.

Conjugated bile acids activate the extracellular regulated protein kinase (ERK1/2) and protein kinase B (Akt) signaling pathways via the sphingosine-1-phosphate receptor (SIPR2) [74]. The S1PR2 is expressed in hippocampal pyramidal/granular neurons, and mice lacking this receptor have a high rate of spontaneous seizures and cognitive deficits [75]. SIPR2 activation is a key regulator of sphingosine kinase 2 (SphK2) [74], which phosphorylates sphingosine to sphingosine-1-phosphate, resulting in increased levels of BDNF and neurogenesis [76]. In this study, healthy volunteers receiving ketamine had increased CSF concentrations of SM 18:0, SM18:1, SM 16:0, and SM 24:1. Increases in SMs would likely be beneficial, given that lower circulating levels of SM have been reported in MDD patients [77], and circulating levels of SM C18:1 were increased in MDD patients post-ketamine [35]. In mouse plasma, only SM C24:0 was increased in (2 *R*,6 *R*)-HNK-treated mice. A similar increase of SMC24:0 was also observed following treatment with the SSRIs citalopram and escitalopram [78]. This increase may also have resulted from an indirect and/or direct effect on acid sphingomyelinase activity, which catalyzes the degradation of sphingomyelin to phosphorylcholine and ceramide [35]. In contrast, Cer (d18:1/23:0) increased in the plasma of ketamine-treated mice, with Hex Cer (d18:1/24:0) increasing in the hippocampus of both (2 *R*,6 *R*)-HNK and ketamine-treated mice, and HexCer (d18:1/18:0), Cer (d18:1/24:1), Hex Cer (d18:1/20:0), and Hex Cer (d18:1/22:0) increasing in the hippocampus of ketamine-treated mice. While these findings are unexpected, previous studies also found that patients treated with antidepressants had higher ceramide levels than patients not taking these drugs [79]. The results suggest that ketamine plays a role in the “sphingolipid-rheostat” [35, 80]. Notably, changes in the sphingolipid/ceramide profile can alter the organization of specific lipid microdomains, resulting in brain function changes [81].

Lipids carry out a variety of functions, including membrane formation and trafficking, storing energy, neuronal signaling and survival, sub-compartmentalizing cell membranes, forming functional platforms that operate in signaling, acting as second messengers in signal transduction, and regulating glucocorticoid action as well as inflammatory processes [82]. In this study, several LPCs increased in the plasma of healthy volunteers post-ketamine, including LPC a C18:2, which at low circulating levels has been linked to impaired glucose tolerance, insulin resistance, type 2 diabetes, memory impairment, and coronary heart disease [83]. Similar increases were found in mouse hypothalamus in both ketamine- and (2 *R*,6 *R*)-HNK-treated mice, including increased LPC a C18:1 in ketamine-treated mice. These increases could be anti-inflammatory and/or could indicate increased mitochondrial oxidative capacity, given that LPCs are precursors for cardiolipin [84]. The large reduction in FA 18:2 plasma levels in ketamine-treated healthy volunteers may also have anti-inflammatory effects by reducing the availability of FA 18:2 metabolism to arachidonic acid [85].

Several PCs contributed positively to PC2 in the CSF of healthy volunteers. In the mouse arm of the study, several PCs increased following either and/or both treatments in plasma, with several of the PCs circulating in mouse plasma containing a saturated FA; in our study, this was predominantly eicosanoic acid. The changes in PC levels may have resulted from the remodeling of the PC species, a combination of effect on phospholipase and/or acyltransferase activity [78], consistent with a study reporting that ketamine affected phospholipase activity [86]. A similar increase in PC levels was observed after treatment with the SSRIs, citalopram and escitalopram [78]. An increase in circulating concentrations of ether-phospholipids was also observed, which is believed to be beneficial because reduced levels of ether-lipids reduce brain levels of various neurotransmitters [87]. Interestingly, in the hypothalamus, PC ae C36:5 decreased following either ketamine or (2 *R*,6 *R*)-HNK treatment, and PC aa C36:6 also decreased following (2 *R*,6 *R*)-HNK treatment. A reduction in the relative abundance of several PC species in the prefrontal cortex of rats was found after administration of the antidepressants maprotiline and paroxetine [82, 88]. Paroxetine also increased levels of cholesterol and TGs, whereas fluoxetine decreased cholesterol and TG levels [89]. In our study, most TGs were elevated following ketamine treatment in the plasma of healthy volunteers. While a similar observation was made in ketamine-treated mice for three TGs, four TGs were decreased in (2 *R*,6 *R*)-HNK-treated mice, demonstrating possible distinct effects of (2 *R*,6 *R*)-HNK and ketamine and suggesting differential mechanisms.

Similar metabolomic changes between treatments could be attributed to the metabolism of ketamine to (2 *R*,6 *R*)-HNK, while treatment-specific changes could help elucidate the mechanism of action underlying both ketamine and (2 *R*,6 *R*)-HNK. In addition to the treatment-specific changes discussed above, in comparative analysis between ketamine and (2 *R*,6 *R*)-HNK, ketamine-treated mice also had decreased circulating levels of the excitatory amino acid neurotransmitters glutamate and aspartate, indicating NMDA-R dependent effects. However, similar effects were not observed in (2 *R*,6 *R*)-HNK treated mice, where increased circulating levels of serotonin were noted instead. The changes in circulating metabolite levels, as well as region-specific changes in the brain, suggest a different mechanism of action for (2 *R*,6 *R*)-HNK than ketamine. Notably, however, the region-specific changes are consistent with recent reports demonstrating that metabolite concentrations differ selectively in discrete regions of the brain [90, 91].

The present study has several strengths. In particular, participants were drug-free prior to the experiment, and the corresponding CSF and plasma were collected from each participant at multiple timepoints, and with minimum invasiveness after the initial intrathecal catheter insertion. Furthermore, the mouse arm of the study, carried out in parallel, offered insight into the contribution of (2 *R*,6 *R*)-HNK to the changes in the ketamine metabolome. Nevertheless, the study is also associated with several limitations, particularly the limited sample size. In addition, ketamine’s effects on healthy volunteers may differ from what would be observed in a patient sample. Finally, because this was an exploratory study, there is the possibility of Type I error (see rationale for analytic plan in the Supplementary Materials); future prospective studies should evaluate the replicability of the conclusions drawn from these results.

## Conclusion

Inflammation has been associated with the pathogenesis of MDD [34, 92]. The present study demonstrated that ketamine and/or (2 *R*,6 *R*)-HNK affect multiple pathways associated with inflammatory conditions (Fig. 3). Interestingly, circulating plasma levels of the metabolites did not always mirror circulating CSF levels, suggesting compartmental-specific changes between plasma and CSF. Furthermore, region-selective differences in metabolite concentrations were observed in the hippocampus and/or hypothalamus of mice. Several metabolic changes following ketamine treatment were unique to either the healthy human volunteer and/or the mouse arm of the study; thus suggesting that different pathways may be differentially involved in ketamine’s antidepressant effects. Understanding such interconnections may be important for improving our understanding of the pathophysiology of depression as well as for refining new therapeutic strategies. Nevertheless, it should be noted that several aspects were consistent between the human metabolome in plasma and CSF and the mouse arm of the study; these include the potential roles of LAT1, IDO1, NAD^+^, the NO signaling pathway, and sphingolipid rheostat in the mechanisms underlying ketamine’s and/or (2 *R*,6 *R*)-HNK’s mechanism of action (Fig. 3). Future studies may wish to explore the role of LAT1 inhibitors, IDO1 inhibitors, and NAMPT inhibitors on the effects of ketamine and/or (2 *R*,6 *R*)-HNK.

## Supplementary information

Suppl Methods

Suppl Figure S1

Suppl Table S1

Suppl Table S2

Suppl Table S3

Suppl Table S4

Suppl Table S5

Suppl Table S6

Suppl Table S7

Suppl Table S8

## References

[1] Dundee JW, Knox JW, Black GW, Moore J, Pandit SK, Bovill J (1970). Ketamine as an induction agent in anaesthetics. Lancet.

[2] Berman RM, Cappiello A, Anand A, Oren DA, Heninger GR, Charney DS (2000). Antidepressant effects of ketamine in depressed patients. Biol Psychiatry.

[3] Zarate CA, Singh JB, Carlson PJ, Brutsche NE, Ameli R, Luckenbaugh DA (2006). A randomized trial of an N-methyl-D-aspartate antagonist in treatment-resistant major depression. Arch Gen Psychiatry.

[4] Zarate CA, Brutsche N, Laje G, Luckenbaugh DA, Venkata SLV, Ramamoorthy A (2012). Relationship of ketamine’s plasma metabolites with response, diagnosis, and side effects in major depression. Biol Psychiatry.

[5] Chong C, Schug SA, Page-Sharp M, Jenkins B, Ilett KF (2009). Development of a sublingual/oral formulation of ketamine for use in neuropathic pain: Preliminary findings from a three-way randomized, crossover study. Clin Drug Investig.

[6] Goldberg ME, Torjman MC, Schwartzman RJ, Mager DE, Wainer IW (2010). Pharmacodynamic profiles of ketamine (R)- and (S)- with 5-day inpatient infusion for the treatment of complex regional pain syndrome. Pain Physician.

[7] Moaddel R, Venkata SLV, Tanga MJ, Bupp JE, Green CE, Iyer L (2010). A parallel chiral–achiral liquid chromatographic method for the determination of the stereoisomers of ketamine and ketamine metabolites in the plasma and urine of patients with complex regional pain syndrome. Talanta.

[8] Gao M, Rejaei D, Liu H (2016). Ketamine use in current clinical practice. Acta Pharm Sin.

[9] Feder A, Costi S, Rutter SB, Collins AB, Govindarajulu U, Jha MK (2021). A randomized controlled trial of repeated ketamine administration for chronic posttraumatic stress disorder. Am J Psych.

[10] Pradhan B, Mitrev L, Moaddel R, Wainer IW (2018). d-Serine is a potential biomarker for clinical response in treatment of post-traumatic stress disorder using (R,S)-ketamine infusion and TIMBER psychotherapy: A pilot study. Biochim Biophys Acta.

[11] Clements JA, Nimmo WS (1981). Pharmacokinetics and analgesic effect of ketamine in man. Br J Anaesth.

[12] Roytblat L, Talmor D, Rachinsky M, Greemberg L, Pekar A, Appelbaum A (1998). Ketamine attenuates the interleukin-6 response after cardiopulmonary bypass. Anesth Analg.

[13] Krystal JH, Karper LP, Seibyl JP, Freeman GK, Delaney R, Bremner JD (1994). Subanesthetic effects of the noncompetitive NMDA antagonist, ketamine, in humans. Arch Gen Psychiatry.

[14] Morgan CJ, Curran HV (2012). Independent Scientific Committee on Drugs. Ketamine use: A review. Addiction.

[15] Zanos P, Moaddel R, Morris PJ, Riggs LM, Highland JN, Georgiou P (2018). Ketamine and ketamine metabolite pharmacology: insights into therapeutic mechanisms. Pharm Rev.

[16] Monteggia LM, Gideons E, Kavalali ET (2013). The role of eukaryotic elongation factor 2 kinase in rapid antidepressant action of ketamine. Biol Psychiatry.

[17] Carvajal FJ, Mattison HA, Cerpa W. Role of NMDA receptor-mediated glutamatergic signaling in chronic and acute neuropathologies. Neural Plast. 2016 10.1155/2016/2701526. 10.1155/2016/2701526 PMC5007376 27630777

[18] Sleigh J, Harvey M, Voss L, Denny B (2014). Ketamine – more mechanisms of action than just NMDA blockade. Trends Anaesth Crit Care.

[19] Witkin JM, Monn JA, Schoepp DD, Li X, Overshiner C, Mitchell SN (2016). The rapidly acting antidepressant ketamine and the mGlu2/3 receptor antagonist LY341495 rapidly engage dopaminergic mood circuits. J Pharm Exp Ther.

[20] Faccio AT, Ruperez FJ, Singh NS, Angulo S, Tavares MFM, Bernier M (2018). Stereochemical and structural effects of (2*R*,6*R*)-hydroxynorketamine on the mitochondrial metabolome in PC-12 cells. Biochim Biophys Acta.

[21] Fukumoto K, Fogaça MV, Liu RJ, Duman C, Kato T, Li XY (2019). Activity-dependent brain-derived neurotrophic factor signaling is required for the antidepressant actions of (2*R*,6*R*)-Hydroxynorketamine. Proc Natl Acad Sci USA.

[22] Schwenk ES, Torjman MC, Moaddel R, Lovett J, Katz D, Denk W, et al. Ketamine for refractory chronic migraine: An observational pilot study and metabolite analysis. J Clin Pharm. 2021 10.1002/jcph.1920. 10.1002/jcph.1920 PMC8769496 34125442

[23] Zanos P, Moaddel R, Morris P, Georgiou P, Fischell J, Elmer GI (2016). NMDAR inhibition-independent antidepressant actions of ketamine metabolites. Nature.

[24] Chou D, Peng HY, Lin TB, Lai CY, Hsieh MC, Wen YC (2018). 2R,6R)-hydroxynorketamine rescues chronic stress-induced depression-like behavior through its actions in the midbrain periaqueductal gray. Neuropharmacology.

[25] Pham TH, Defaix C, Xu X, Deng SX, Fabresse N, Alvarez JC (2018). Common neurotransmission recruited in (R,S)-ketamine and (2*R*,6*R*)-hydroxynorketamine-induced sustained antidepressant-like effects. Biol Psychiatry.

[26] Lumsden EW, Troppoli TA, Myers SJ, Zanos P, Aracava Y, Kehr J (2019). Antidepressant-relevant concentrations of the ketamine metabolite (2*R*,6*R*)-hydroxynorketamine do not block NMDA receptor function. Proc Natl Acad Sci USA.

[27] Zanos P, Highland JN, Stewart BW, Georgiou P, Jenne CE, Lovett J (2019). 2R,6R)-hydroxynorketamine exerts mGlu2 receptor-dependent antidepressant actions. Proc Natl Acad Sci USA.

[28] Zanos P, Highland JN, Liu X, Troppoli TA, Georgiou P, Lovett J (2019). R)-ketamine exerts antidepressant actions partly via conversion to (2R,6R)-hydroxynorketamine, while causing adverse effects at sub-anaesthetic doses. Br J Pharmacol.

[29] Highland JN, Morris PJ, Zanos P, Lovett J, Ghosh S, Wang A (2019). Mouse, rat, and dog bioavailability and mouse oral antidepressant efficacy of (2*R*,6*R*)-hydroxynorketamine. J Psychopharmacol.

[30] Aguilar-Valles A, De Gregorio D, Matta-Camacho E, Eslamizade MJ, Khlaifia A, Skaleka A (2021). Antidepressant actions of ketamine engage cell-specific translation via eIF4E. Nature.

[31] Chen BK, Luna VM, LaGamma CT, Xu X, Deng SX, Suckow RF (2020). Sex-specific neurobiological actions of prophylactic (R,S)-ketamine, (2*R*,6*R*)-hydroxynorketamine, and (2S,6S)-hydroxynorketamine. Neuropsychopharmacology.

[32] Kroin JS, Das V, Moric M, Buvanendran A (2019). Efficacy of the ketamine metabolite (2*R*,6*R*)-hydroxynorketamine in mice models of pain. Reg Anesth Pain Med.

[33] Shaffer CL, Dutra JK, Tseng WC, Weber ML, Bogart LJ, Hales K (2019). Pharmacological evaluation of clinically relevant concentrations of (2*R*,6*R*)-hydroxynorketamine. Neuropharmacology.

[34] Johnson C, Ivanisevic J, Siuzdak G (2016). Metabolomics: Beyond biomarkers and towards mechanisms. Nat Rev Mol Cell Biol.

[35] Moaddel R, Shardell M, Khadeer M, Lovett J, Kadriu B, Ravichandran S (2018). Plasma metabolomic profiling of a ketamine and placebo crossover trial of major depressive disorder and healthy control subjects. Psychopharmacology.

[36] Zhang M, Wen C, Zhang Y, Sun F, Wang S, Ma J (2015). Serum metabolomics in rats models of ketamine abuse by gas chromatography-mass spectrometry. J Chromatogr B Anal Technol Biomed Life Sci.

[37] Wen C, Zhang M, Zhang Y, Sun F, Ma J, Hu L (2015). Brain metabolomics in rats after administration of ketamine. Biomed Chromatogr.

[38] Demarest TG, Truong GTD, Lovett J, Mohanty JG, Mattison JA, Mattson MP (2019). Assessment of NAD+ metabolism in human cell cultures, erythrocytes, cerebrospinal fluid and primate skeletal muscle. Anal Biochem.

[39] McGarry A, Gaughan J, Hackmyer C, Lovett J, Khadeer M, Shaikh H, et al. Cross-sectional analysis of plasma and CSF metabolomic markers in Huntington’s disease for participants of varying functional disability: A pilot study. Sci Rep. 2020 10.1038/s41598-020-77526-9. 10.1038/s41598-020-77526-9 PMC7686309 33235276

[40] Lautrup S, Sinclair DA, Mattson MP, Fang EF (2019). NAD+ in brain aging and neurodegenerative disorders. Cell Metab.

[41] Zukunft S, Prehn C, Röhring C, Möller G, Hrabӗ de Angelis M, Adamski J, et al. High-throughput extraction and quantification method for targeted metabolomics in murine tissues. Metabolomics 2018 10.1007/s11306-017-1312-x. 10.1007/s11306-017-1312-x PMC5748028 29354024

[42] Westbrook R, Chung T, Lovett J, Ward C, Joca H, Yang H, et al. Kynurenines link chronic inflammation to functional decline and physical frailty. JCI Insight. 2020 10.1172/jci.insight.136091. 10.1172/jci.insight.136091 PMC7455140 32814718

[43] Wasserstein RL, Schirm AL, Lazar NA. Moving to a world beyond “p < 0.05”. American Stat. 2019 10.1080/00031305.2019.1583913.

[44] Jaki T, Wolfsegger MJ (2011). Estimation of pharmacokinetic parameters with the R package PK. Pharm Stat.

[45] Li N, Lee B, Liu RJ, Banasr M, Dwyer JM, Iwata M (2010). mTOR-dependent synapse formation underlies the rapid antidepressant effects of NMDA antagonists. Science.

[46] Ignácio ZM, Réus GZ, Arent CO, Abelaira HM, Pitcher MR, Quevedo J (2016). New perspectives on the involvement of mTOR in depression as well as in the action of antidepressant drugs. Br J Clin Pharm.

[47] Baranyi A, Amouzadeh-Ghadikolai O, von Lewinski D, Rothenhӓusler HB, Theokas S, Robier C, et al. Branched-chain amino acids as new biomarkers of major depression - a novel neurobiology of mood disorder. PLoS One. 2016 10.1371/journal.pone.0160542. 10.1371/journal.pone.0160542 PMC4973973 27490818

[48] Scalise M, Galluccio M, Console L, Pochini L, Indiveri C. The human SLC7A5 (LAT1): The intriguing histidine/large neutral amino acid transporter and its relevance to human health. Front Chem. 2018 10.3389/fchem.2018.00243. 10.3389/fchem.2018.00243 PMC6023973 29988369

[49] Walker AK, Wing EE, Banks WA, Dantzer R (2019). Leucine competes with kynurenine for blood-to-brain transport and prevents lipopolysaccharide-induced depression-like behavior in mice. Mol Psychiatry.

[50] Miller AH (2013). Conceptual confluence: The kynurenine pathway as a common target for ketamine and the convergence of the inflammation and glutamate hypotheses of depression. Neuropsychopharmacology.

[51] Heisler JM, O’Connor JC (2015). Indoleamine 2,3-dioxygenase-dependent neurotoxic kynurenine metabolism mediates inflammation-induced deficit in recognition memory. Brain Behav Immun.

[52] Dobos N, de Vries EF, Kema IP, Patas K, Prins M, Nijholt IM (2012). The role of indoleamine 2,3-dioxygenase in a mouse model of neuroinflammation-induced depression. J Alzheimers Dis.

[53] Fricker AR, Green LE, Jenkins SI, Griffin SM. The influence of nicotinamide on health and disease in the central nervous system. Int J Tryptophan Res. 2018 10.1177/1178646918776658. 10.1177/1178646918776658 PMC5966847 29844677

[54] Ima IS, Guarente L (2014). NAD+ and sirtuins in aging and disease. Trends Cell Biol.

[55] Herskovits A, Guarente L (2013). Sirtuin deacetylases in neurodegenerative diseases of aging. Cell Res.

[56] Friedrich H, Adriouch S, Braß A, Jung C, Möller S, Scheuplein F (2007). Extracellular NAD and ATP: Partners in immune cell modulation. Purinergic Signal.

[57] Valentina A, Gianluca MV, Silvia D NAMPT and NAPRT: Two metabolic enzymes with key roles in inflammation. Front Oncol. 2020 10.3389/fonc.2020.00358. 10.3389/fonc.2020.00358 PMC7096376 32266141

[58] Busso N, Karababa M, Nobile M, Rolaz A, van Gool F, Galli M, et al. Pharmacological inhibition of nicotinamide phosphoribosyltransferase/visfatin enzymatic activity identifies a new inflammatory pathway linked to NAD. PLoS One 2008 10.1371/journal.pone.0002267. 10.1371/journal.pone.0002267 PMC2377336 18493620

[59] Wu GC, Liao WI, Wu SY, Pao HP, Tang SE, Li MH, et al. Targeting of nicotinamide phosphoribosyltransferase enzymatic activity ameliorates lung damage induced by ischemia/reperfusion in rats. Respir Res. 2017 10.1186/s12931-017-0557-2. 10.1186/s12931-017-0557-2 PMC5404693 28438162

[60] Hwang ES, Song SB. Possible adverse effects of high-dose nicotinamide: Mechanisms and safety assessment. Biomolecules 2020 10.3390/biom10050687. 10.3390/biom10050687 PMC7277745 32365524

[61] Liu Z, Li C, Fan X, Kuang Y, Zhang X, Chen L, et al. Nicotinamide, a vitamin B3 ameliorates depressive behaviors independent of SIRT1 activity in mice. Mol Brain. 2020 10.1186/s13041-020-00703-4. 10.1186/s13041-020-00703-4 PMC7686777 33228716

[62] Wu J, Kikuchi T, Wang Y, Sato K, Fukuyama F, Sakaii M, et al. Ketamine can induce the increase of NOx- level in the hippocampus by activating NOS but not via glutamate receptors: in vivo study with microdialysis in rats. Anesthesia Analgesia. 1999 10.1097/00000539-199902001-00399.

[63] Bevilacqua L, Charney A, Pierce CR, Richards SM, Jha MK, Glasgow A (2021). Role of nitric oxide signaling in the antidepressant mechanism of action of ketamine: A randomized controlled trial. J Psychopharmacol.

[64] Lara N, Archer SL, Baker GB, Le Melledo JM (2003). Paroxetine-induced increase in metabolic end products of nitric oxide. J Clin Psychopharm.

[65] Li T, Chiang JYL. Regulation of bile acid and cholesterol metabolism by PPARs. PPAR Res. 2009 10.1155/2009/501739. 10.1155/2009/501739 PMC2712638 19636418

[66] Varma VR, Wang Y, An Y, Varma S, Bilgel M, Doshi J, et al. Bile acid synthesis, modulation, and dementia: a metabolomic, transcriptomic, and pharmacoepidemiologic study. PLoS Med. 2021 10.1371/journal.pmed.1003615. 10.1371/journal.pmed.1003615 PMC8158920 34043628

[67] Monteiro-Cardoso VF, Corlianò M, Singaraja RR (2021). Bile acids: A communication channel in the gut-brain axis. Neuromolecular Med.

[68] Ferrell JM, Chiang JYL (2021). Bile acid receptors and signaling crosstalk in the liver, gut and brain. Liver Res.

[69] Koch A, Bonus M, Gohlke H, Klocker N. Isoform-specific inhibition of N-methyl-D-aspartate receptors by bile salts. Sci Rep. 2019 10.1038/s41598-019-46496-y. 10.1038/s41598-019-46496-y PMC6624251 31296930

[70] Cartocci V, Servadio M, Trezza V, Pallottini V (2017). Can Cholesterol metabolism modulation affect brain function and behavior?. J Cell Physiol.

[71] Segatto M, Di Giovanni A, Marino M, Pallottini V (2013). Analysis of the protein network of cholesterol homeostasis in different brain regions: an age and sex dependent perspective. J Cell Physiol.

[72] Moaddel R, Sanghvi S, Dossou KS, Ramamoorthy A, Green C, Bupp J, et al. The distribution and clearance of (2S,6S)-hydroxynorketamine, an active ketamine metabolite, in Wistar rats. Pharmacol Res Perspect. 2015 10.1002/prp2.157. 10.1002/prp2.157 PMC4492732 26171236

[73] Casarotto PC, Girych M, Fred SM, Kovaleva V, Moliner R, Enkavi G (2021). Antidepressant drugs act by directly binding to TRKB neurotrophin receptors. Cell.

[74] Kwong E, Li Y, Hylemon PB, Zhou H (2015). Bile acids and sphingosine-1-phosphate receptor 2 in hepatic lipid metabolism. Acta Pharm Sin B.

[75] Muller CP, Reichel M, Muhle C, Rhein C, Gulbins E, Kornhuber J (2015). Brain membrane lipids in major depression and anxiety disorders. Biochim Biophys Acta.

[76] Anderson G, Maes M (2014). Reconceptualizing adult neurogenesis: Role for sphingosine-1-phosphate and fibroblast growth factor-1 in co-ordinating astrocyte-neuronal precursor interactions. CNS Neurol Disord Drug Targets.

[77] Kornhuber J, Medlin A, Bleich S, Jendrossek V, Henkel AW, Wiltfang J (2005). High activity of acid sphingomyelinase in major depression. J Neural Transm.

[78] MahmoudianDehkordi S, Ahmed AT, Bhattacharyya S, Han X, Baillie RA, Arnold M, et al. Alterations in acylcarnitines, amines, and lipids inform about the mechanism of action of citalopram/escitalopram in major depression. Transl Psychiatry. 2021 10.1038/s41398-020-01097-6. 10.1038/s41398-020-01097-6 PMC7925685 33654056

[79] Brunkhorst-Kanaan N, Klatt-Schreiner K, Hackel J, Schröter K, Trautmann S, Hahnefeld L (2019). Targeted lipidomics reveal derangement of ceramides in major depression and bipolar disorder. Metabolism.

[80] Van Brocklyn JR, Williams JB (2012). The control of the balance between ceramide and sphingosine-1-phosphate by sphingosine kinase: oxidative stress and the seesaw of cell survival and death. Comp Biochem Physiol B Biochem Mol Biol.

[81] Oliveira TG, Chan RB, Bravo FV, Miranda A, Silva RR, Zhou B (2016). The impact of chronic stress on the rat brain lipidome. Mol Psychiatry.

[82] Walther A, Cannistraci CV, Simons K, Duran C, Mathias JG, Wehrli S, et al. Lipidomics in major depressive disorder. Front Psychiatry. 2018 10.3389/fpsyt.2018.00459. 10.3389/fpsyt.2018.00459 PMC6196281 30374314

[83] Gonzalez-Freire M, Moaddel R, Sun K, Fabbri E, Zhang P, Khadeer M (2019). Targeted metabolomics shows low plasma lysophosphatidylcholine 18:2 predicts greater decline of gait speed in older adults: The baltimore longitudinal study of aging. J Gerontol A Biol Sci Med Sci.

[84] Semba RD, Moaddel R, Zhang P, Ramsden CE, Ferrucci L (2019). (2019) Tetra-linoleoyl cardiolipin depletion plays a major role in the pathogenesis of sarcopenia. Med Hypotheses.

[85] Innes JK, Calder PC (2018). Omega-6 fatty acids and inflammation. Prostaglandins Leukot Ess Fat Acids.

[86] Denson DD, Worrell RT, Eaton DC. A possible role for phospholipase A2 in the action of general anesthetics. Am J Physiol. 1996 10.1152/ajpcell.1996.270.2.C636. 10.1152/ajpcell.1996.270.2.C636 8779929

[87] Dorninger F, Gundacker A, Zeitler G, Pollak DD, Berger J. Ether lipid deficiency in mice produces a complex behavioral phenotype mimicking aspects of human psychiatric disorders. Int J Mol Sci. 2019 10.3390/ijms20163929. 10.3390/ijms20163929 PMC6720005 31412538

[88] Wray NH, Rasenick MM (2019). NMDA-receptor independent actions of ketamine: A new chapter in a story that’s not so old. Neuropsychopharmacology.

[89] Olguner Eker Ö, Özsoy S, Eker B, Doğan H (2017). Metabolic effects of antidepressant treatment. Noro Psikiyatr Ars.

[90] Heeley N, Blouet C. Central amino acid sensing in the control of feeding behavior. Front Endocrinol. 2016 10.3389/fendo.2016.00148. 10.3389/fendo.2016.00148 PMC5120084 27933033

[91] Kahn B, Myers M (2006). mTOR tells the brain that the body is hungry. Nat Med.

[92] Kiecolt-Glaser J, Derry HM, Fagundes CP (2015). Inflammation: Depression fans the flames and feasts on the heat. Am J Psych.

