# Supplementary material for: Comparative metabolomic analysis in plasma and cerebrospinal fluid of humans and in plasma and brain of mice following antidepressant-dose ketamine administration
Source: Transl Psychiatry. 2022 May 2;12:179. doi: 10.1038/s41398-022-01941-x (PMC9061764; doi:10.1038/s41398-022-01941-x)
Supplement: Supplementary file 1 — Suppl Methods [file 41398_2022_1941_MOESM1_ESM.docx]

*Moaddel et al —* *Comparative Metabolomic Analysis in Plasma and Cerebrospinal Fluid in Humans and in Plasma and Brain in Mice Following Antidepressant-dose Ketamine Administration*

**SUPPLEMENTARY METHODS**

*Rationale for analytic approach*

Because the number of potentially interesting comparisons in this study were a function of the large number of features involved in any metabolomic analysis, the multiple timepoints, and the multiple groups (in the case of the mouse data), the use of *p*-values to flag results based on “statistical significance” would have resulted in an unacceptably high rate of Type I error. One approach to address this would be to account for the familywise error rate, such as in FDR-corrected *p-*values, although the definition of “family” in this study is unclear. However, it should be noted that the binarization of *p*-values is misleading because it homogenizes very dissimilar values (e.g., 0.10 and 0.90) and yet treats substantively identical values as qualitatively different (e.g., 0.049 and 0.051).

This exploratory analysis did not expect to draw discrete decisions about each feature but rather to understand the strength of the evidence in its favor. Still, given the number of features, a method to reduce the number of candidates worthy of further attention needed to be used. Thus, for both the human and mouse data, an initial analysis (described below) was performed to identify features of interest, relying on the raw *p*-values as an index. Once these features were identified, a more qualitative approach was taken to evaluate the strength of the evidence in favor of the feature’s relevance to ketamine’s mechanism of action. Here, current American Statistical Association guidelines [1] were followed in reporting uncorrected *p*-values focusing on the magnitude and variability (confidence intervals) in effects. Contextual information was also considered, such as the timing and persistence of the effects (e.g., a difference observed at a single timepoint holds less weight than a consistent pattern of differences across timepoints) and the behavior of other related features. This process, like all statistical analyses, was inherently subjective [2]. Therefore, parameter estimates, test statistics, and *p*-values for all comparisons are also provided so that interested researchers may form their own interpretations.

*Metabolomics Panels*

*Metabolomic Assay:* A targeted metabolomic assessment was carried out following the manufacturer’s protocol using Biocrates’ commercially available MxP® Quant 500 kit (Biocrates, Innsbruck, Austria), which quantifies 630 distinct metabolites in human and rodent plasma (10 µl), human CSF (10 µl), and mouse brain (10 µl). For the mouse hypothalamus and hippocampus, tissue was homogenized following a previously published protocol with slight modifications [3]. Briefly, frozen tissue was suspended in ice cold 85% ethanol/15% phosphate buffer [10mM, pH 7.5] in a 1:6 ratio (1 mg tissue/µl solvent) and homogenized using the Precellys Lysing Kit CKMix_WP 96 preps (Bertin Technologies, France) on a Precellys Evolution (Bertin Technologies, France) twice for 30 seconds at 6000 rpm with 30-second pause intervals. After homogenization, the samples were centrifuged at 3200×g for three minutes, and 10 µl of the supernatant was used for metabolomic analysis. Concentrations were calculated using the Analyst/MetIDQ software and reported in µmol/L. Data were quantified using appropriate mass spectrometry software (Sciex Analyst®) and imported into Biocrates MetIDQ™ software for further analysis. The data were target-normalized to internal quality controls from Biocrates. Pooled samples were repeated across plates to ensure accuracy of the measurements across plates.

*NAD^+^ metabolome*: In the human studies, 20 μl of CSF was solubilized in 60 μl of methanol, including the addition of 5 μl of internal standard. For the mouse studies, 15 μl of whole blood was solubilized in 135 μl of buffered ethanol [75% ethanol/25% 1 mM HEPES, pH 7.1] including 10 μl of internal standard. Samples were then centrifuged at 4°C for 10 minutes at 13,200×g to remove the protein pellet. The supernatant was collected and placed in an autosampler vial for analysis as previously described [4,5]. Briefly, the NAD^+^ metabolites were resolved using an Accucore HILIC column (2.1 × 150 mm, 2.6 μm, Thermo) at 32 °C. Ammonium acetate [7.5 mM, pH 7.86] was used as mobile phase A and acetonitrile as mobile phase B with a 10 µl injection volume. The following linear gradient was run for 14.0 minutes at a flow rate of 0.4 ml/min: 0–1 min 90% B, 1.5 min 72.5% B, 2.5 min 67.5% B, 8.0 min 20% B, 10 min 20% B, 10.1 min 90% B. Several ratios were also considered including nicotinamide mononucleotide (NMN):nicotinamide (NAM) for nicotinamide phosphoribosyltranserase (NAMPT), nicotinamide adenine dinucleotide phosphate (NADP):reduced nicotinamide adenine dinucleotide phosphate (NADPH) for nicotinamide nucleotide transhydrogenase (NNT)and Glucose-6-phosphate dehydrogenase (G6PDH), NADP/NAD^+^ for NAD Kinase (NADK), NAD^+^/NMN for nicotinamide mononucleotide adenylyl transferases (NMNATs) and NAD^+^/NADH for dehydrogenases (DHs).

**References**

[1] Wasserstein RL, Schirm AL, Lazar NA. Moving to a world beyond “p < 0.05”. *The American Statistician* <https://doi.org/10.1080/00031305.2019.1583913> (2019).

[2] Berger JO, Berry DA. Statistical Analysis and the Illusion of Objectivity. *American Scientist* 1988; 76(2): 159-165.

[3] Zukunft S, Prehn C, Röhring C, Möller G, Hrabӗ de Angelis M, Adamski J *et al*. High-throughput extraction and quantification method for targeted metabolomics in murine tissues. *Metabolomics* <https://doi.org/10.1007/s11306-017-1312-x> (2018).

[4] Demarest TG, Truong GTD, Lovett J, Mohanty JG, Mattison JA, Mattson MP *et al*. Assessment of NAD+ metabolism in human cell cultures, erythrocytes, cerebrospinal fluid and primate skeletal muscle. *Anal Biochem* 2019; 572: 1-8.

[5] McGarry A, Gaughan J, Hackmyer C, Lovett J, Khadeer M, Shaikh H *et al*. Cross-sectional analysis of plasma and CSF metabolomic markers in Huntington’s disease for participants of varying functional disability: a pilot study. *Sci Rep* <https://doi.org/10.1038/s41598-020-77526-9> (2020).

**LIST OF SUPPLEMENTARY FIGURES AND TABLES**

Supplementary Figure S1. Change in metabolome principal components (PCs) over time. Metabolomic data were normalized to baseline and then underwent a principal components analysis (PCA). The mean of each component over time was estimated using a linear mixed model and plotted with its 95% confidence interval. Left panel: Plasma data. Right panel: CSF data. See Supplemental Table S2 for comprising features.

Supplementary Table S1. Demographic information for human participants

Supplementary Table S2. **Strongest loadings for all principal components analysis (PCA) components in healthy human plasma and CSF metabolomic data.** Feature name and loading for strongest 20 loadings on each component from the PCA of human plasma and CSF metabolomic features.

Supplementary Table S3. **Univariate analysis of plasma metabolites.** See Excel file for table. Values were Pareto-scaled to baseline and subjected to a linear mixed effects model. Table contains degrees of freedom, test statistics, and uncorrected *p*-values for the test of the difference from zero (baseline) in the estimated marginal mean at each post-infusion timepoint. Negative test statistics reflect a decrease from baseline; positive test statistics reflect an increase from baseline.

Supplementary Table S4. **Univariate analysis of CSF metabolites.** See Excel file for table. Values were Pareto-scaled to baseline and subjected to a linear mixed effects model. Table contains degrees of freedom, test statistics, and uncorrected *p*-values for the test of the difference from zero (baseline) in the estimated marginal mean at each post-infusion timepoint. Negative test statistics reflect a decrease from baseline; positive test statistics reflect an increase from baseline.

Supplementary Table S5. **Univariate analysis of mouse plasma AUC.** See Excel file for table. The Area Under the Curve (AUC) calculated for serial sacrificed design of each metabolite was compared using a Z-transformation between groups receiving 10 mg/kg ketamine, 10 mg/kg (2*R*,6*R*)-hydroxynorketamine (HNK), or saline in mouse plasma. Table shows test statistics (Z) for the group comparisons, along with uncorrected *p*-values. Positive Z-statistics reflect higher values in the treated group relative to saline; negative Z-statistics reflect lower values in the treated group than in saline.

Supplementary Table S6. **Univariate analysis of mouse hippocampus AUC.** See Excel file for table. The Area Under the Curve (AUC) calculated for serial sacrificed design of each metabolite was compared using a Z-transformation between groups receiving 10 mg/kg ketamine, 10 mg/kg (2*R*,6*R*)-hydroxynorketamine (HNK), or saline in mouse hippocampus. Table shows test statistics (Z) for the group comparisons, along with uncorrected *p*-values. Positive Z-statistics reflect higher values in the treated group relative to saline; negative Z-statistics reflect lower values in the treated group than in saline.

Supplementary Table S7. **Univariate analysis of mouse hypothalamus AUC.** See Excel file for table. The Area Under the Curve (AUC) calculated for serial sacrificed design of each metabolite was compared using a Z-transformation, between groups receiving 10 mg/kg ketamine, 10 mg/kg (2*R*,6*R*)-hydroxynorketamine (HNK), or saline in mouse hypothalamus. Table shows test statistics (Z) for the group comparisons, along with uncorrected *p*-values. Positive Z-statistics reflect higher values in the treated group relative to saline; negative Z-statistics reflect lower values in the treated group than in saline.

Supplementary Table S8. **Univariate analysis of mouse plasma nicotinamide adenine dinucleotide** (**NAD^+^) metabolites**. See Excel file for table. Values were Pareto-scaled to the saline group and subjected to a linear mixed effects model. Table contains degrees of freedom, test statistics, and uncorrected *p*-values for the test of the difference between the drug ((2*R*,6*R*)-hydroxynorketamine (HNK) or ketamine (KET)) and saline (SAL) groups in the estimated marginal mean at each timepoint. Negative test statistics reflect a lower value in the drug group relative to saline; positive test statistics reflect a higher value in the dug group relative to saline.
