# Supplementary material for: Comparative metabolomic analysis in plasma and cerebrospinal fluid of humans and in plasma and brain of mice following antidepressant-dose ketamine administration
Source: Transl Psychiatry. 2022 May 2;12:179. doi: 10.1038/s41398-022-01941-x (PMC9061764; doi:10.1038/s41398-022-01941-x)

**Supplemental Figure S1.** Change in metabolome principal components (PCs) over time. Metabolomic data were normalized to baseline and then underwent a principal components analysis (PCA). The mean of each component over time was estimated using a linear mixed model and plotted with its 95% confidence interval. Left panel: Plasma data. Right panel: CSF data. See Supplemental Table S2 for comprising features.


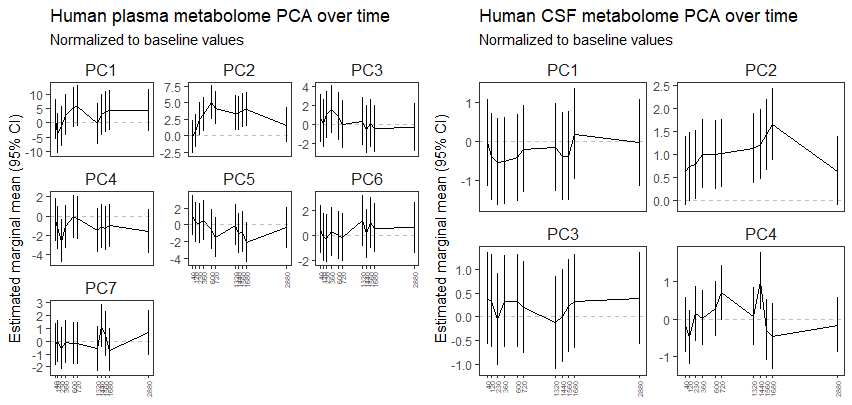

Supplement: Supplementary file 2 — Suppl Figure S1 [file 41398_2022_1941_MOESM2_ESM.docx]
