# Supplementary material for: Comparative metabolomic analysis in plasma and cerebrospinal fluid of humans and in plasma and brain of mice following antidepressant-dose ketamine administration
Source: Transl Psychiatry. 2022 May 2;12:179. doi: 10.1038/s41398-022-01941-x (PMC9061764; doi:10.1038/s41398-022-01941-x)
Supplement: Supplementary file 3 — Suppl Table S1 [file 41398_2022_1941_MOESM3_ESM.docx]

**Supplemental Table S1. Demographic information for human participants**

| **Age (Years)** | **Sex** | **Race** |
| --- | --- | --- |
| 19 | Female | White |
| 21 | Male | Black or African American |
| 23 | Male | Asian |
| 24 | Female | Multiple Races |
| 26 | Female | Black or African American |
| 28 | Male | White |
| 33 | Male | White |
| 36 | Female | White |
| 36 | Male | Black or African American |
