# Supplementary material for: Comparative metabolomic analysis in plasma and cerebrospinal fluid of humans and in plasma and brain of mice following antidepressant-dose ketamine administration
Source: Transl Psychiatry. 2022 May 2;12:179. doi: 10.1038/s41398-022-01941-x (PMC9061764; doi:10.1038/s41398-022-01941-x)
Supplement: Supplementary file 4 — Suppl Table S2 [file 41398_2022_1941_MOESM4_ESM.docx]

**Table S2. Strongest loadings for all principal components analysis (PCA) components in healthy human plasma and CSF metabolomic data.** Feature name and loading for strongest 20 loadings on each component from the PCA of human plasma and CSF metabolomic features.

| **PLASMA** | | | | | | |
| --- | --- | --- | --- | --- | --- | --- |
| **PC1** | **PC2** | **PC3** | **PC4** | **PC5** | **PC6** | **PC7** |
| TG(18:1_30:1) [0.09] | FA(18:2) [-0.24] | Taurine [-0.22] | FA(18:2) [0.25] | TG(22:6_34:2) [0.17] | Taurine [-0.33] | Taurine [0.41] |
| TG(18:2_30:0) [0.09] | GCDCA [0.24] | ProBetaine [-0.18] | C2 [0.17] | TG(22:6_34:1) [0.16] | Histidine [-0.19] | Hippuric Acid [0.19] |
| TG(14:0_36:3) [0.09] | TCDCA [0.2] | Hexose [0.17] | ProBetaine [-0.14] | Taurine [-0.16] | Serine [-0.15] | p-Cresol-SO4 [0.19] |
| TG(18:1_30:2) [0.09] | GDCA [0.17] | Cystine [0.16] | PC aa C36:5 [-0.14] | CE(20:5) [0.15] | HomoArginine [-0.14] | CE(22:5) [0.18] |
| TG(14:0_36:2) [0.09] | beta-Alanine [0.15] | CE(20:4) [0.16] | TG(20:5_36:3) [-0.13] | ProBetaine [-0.14] | PC ae C36:5 [-0.13] | Citrulline [0.17] |
| TG(18:1_30:0) [0.09] | 3-Methyl-Histidine [0.15] | Hippuric Acid [-0.14] | TG(20:4_36:5) [-0.13] | PC aa C38:6 [0.14] | p-Cresol-SO4 [0.12] | Homoarginine [0.17] |
| TG(14:0_34:1) [0.09] | C2 [-0.14] | PC aa C38:4 [0.13] | TG(20:4_32:1) [-0.13] | TG(22:6_32:1) [0.13] | Cystine [0.12] | Arginine [0.15] |
| TG(18:1_32:2) [0.09] | TG(18:0_32:0) [0.14] | PC aa C40:6 [0.12] | TG(20:5_34:2) [-0.12] | PC aa C32:2 [0.13] | beta-Alanine [-0.12] | beta-Alanine [0.14] |
| TG(18:2_34:2) [0.09] | TDCA [0.13] | Glycine [0.12] | TG(18:2_38:6) [-0.12] | TG(16:0_40:8) [0.12] | Citrulline [-0.12] | CE(18:2) [0.14] |
| TG(18:2_32:2) [0.09] | TG(16:0_32:0) [0.13] | LPC a C20:4 [0.12] | TG(17:0_36:3) [0.11] | PC aa C36:6 [0.12] | HexCer(d18:1/24:1) [0.11] | CE(18:1) [0.14] |
| TG(18:2_28:0) [0.09] | GCA [0.13] | Betaine [0.12] | TG(20:5_34:1) [-0.11] | PC aa C36:5 [0.11] | CE(20:3) [0.11] | t4-OH-Pro [-0.13] |
| TG(18:0_36:1) [0.09] | TCA [0.12] | Histidine [-0.12] | TG(18:1_35:2) [0.11] | TG(22:6_34:3) [0.11] | Betaine [-0.11] | SM (OH) C14:1 [0.13] |
| TG(14:0_34:2) [0.09] | TG(18:1_28:1) [0.12] | TG(20:4_36:3) [0.11] | TG(20:4_36:4) [-0.11] | CE(22:6) [0.11] | t4-OH-Pro [-0.11] | 3-IAA [0.13] |
| TG(18:0_36:2) [0.09] | Hippuric Acid [-0.12] | GCDCA [0.11] | PC aa C32:1 [-0.11] | DHA [0.11] | PC aa C36:0 [-0.11] | FA(18:2) [-0.12] |
| TG(16:0_32:2) [0.09] | Methionine [0.12] | LPC C18:2 [0.11] | TG(20:4_32:0) [-0.11] | PC ae C38:0 [0.11] | TG(20:5_36:3) [-0.11] | SM C18:0 [0.12] |
| TG(14:0_36:4) [0.09] | TG(18:0_36:1) [0.12] | SM C18:1 [0.11] | TG(20:4_34:3) [-0.11] | PC ae C34:2 [0.1] | DHEAS [-0.11] | Serine [0.11] |
| TG(18:0_34:2) [0.09] | Phenylalanine [0.12] | TG(20:4_36:4) [0.11] | TG(18:1_34:2) [0.11] | Lysine [0.1] | Sarcosine [-0.11] | Alanine [0.11] |
| TG(18:0_32:2) [0.09] | Met-SO [0.12] | GDCA [0.11] | TG(22:6_32:0) [-0.11] | PC ae C34:1 [0.1] | TG(16:0_32:0) [0.1] | Histidine [0.11] |
| TG(18:0_32:1) [0.09] | TG(18:0_32:2) [0.11] | DHEAS [0.11] | TG(16:0_38:6) [-0.1] | Tryptophan [0.1] | PC aa C36:5 [-0.1] | CE(20:3) [0.11] |
| TG(14:0_36:1) [0.09] | Isoleucine [0.11] | LPC C18:0 [0.1] | TG(16:0_38:7) [-0.1] | PC aa C30:0 [0.1] | GCA [-0.1] | Creatinine [0.11] |
| **CSF** | | | | | | |
| **PC1** | **PC2** | **PC3** | **PC4** |  |  |  |
| t4-OH-Pro [0.28] | PC aa C34:1 [0.33] | Hypoxanthine [0.4] | Betaine [0.43] |  |  |  |
| Valine [0.28] | Glutamate [0.31] | Hexose [0.38] | Arginine [0.41] |  |  |  |
| Leucine [0.26] | SM C16:0 [0.27] | Betaine [-0.38] | Hypoxanthine [0.31] |  |  |  |
| Glycine [0.26] | Tryptophan [-0.25] | Cystine [0.25] | Citrulline [0.27] |  |  |  |
| Isoleucine [0.24] | Citrulline [-0.23] | PC aa C34:1 [-0.24] | t4-OH-Pro [-0.23] |  |  |  |
| Ornithine [0.23] | Creatinine [0.21] | Methionine [0.23] | Xanthine [0.23] |  |  |  |
| Cysteine [0.22] | Betaine [-0.19] | Cysteine [-0.18] | Alanine [0.2] |  |  |  |
| Hypoxanthine [-0.22] | PC aa C32:1 [0.19] | Arginine [0.17] | Threonine [-0.19] |  |  |  |
| Threonine [0.21] | PC aa C36:1 [0.19] | Glutamate [0.16] | Creatinine [0.19] |  |  |  |
| Glutamine [0.19] | PC aa C36:2 [0.18] | SM C18:0 [-0.14] | Serine [0.17] |  |  |  |
| Xanthine [-0.19] | Xanthine [0.18] | ProBetaine [0.14] | Lactic acid [0.16] |  |  |  |
| Alanine [0.18] | SM C18:0 [0.17] | Citrulline [-0.14] | Tyrosine [0.16] |  |  |  |
| TMAO [0.17] | Lactic acid [0.17] | t4-OH-Pro [0.13] | 1-Methyl-Histidine [-0.16] |  |  |  |
| Lysine [0.16] | Arginine [-0.16] | Asparagine [-0.12] | Ornithine [0.14] |  |  |  |
| Methionine [0.16] | PC aa C36:4 [0.15] | SM C16:0 [-0.11] | Phenylalanine [0.13] |  |  |  |
| AABA [0.16] | PC aa C38:4 [0.14] | Xanthine [0.11] | Hexose [0.12] |  |  |  |
| Phenylalanine [0.15] | Threonine [-0.14] | Phenylalanine [-0.11] | Taurine [0.12] |  |  |  |
| 1-Methyl-Histidien [0.14] | Alanine [0.13] | AABA [-0.11] | Glutamate [-0.1] |  |  |  |
| ProBetaine [-0.14] | SDMA [-0.12] | TMAO [-0.1] | Glutamine [0.1] |  |  |  |
| Glutamate [0.13] | SM C24:1 [0.12] | Threonine [0.1] | Glycine [0.1] |  |  |  |

Abbreviations: TG: Triacylglycerol; FA: Fatty acids; LPC: Lysophosphatidylcholine; PC: Phosphatidylcholine; GCDCA: glycochenodeoxycholic acid; CE: Cholesterol Ester; GDCA: glycodeoxycholic acid; TCDCA; taurochenodeoxycholic acid; TDCA: Taurodeoxycholic acid; GCA: glycocholic acid; DHA: Docosahexaenoic acid; DHEAS: Dehydroepiandrosterone sulfate; TMAO: Trimethylamine-N-Oxide; SDMA: Symmetric dimethylarginine; SM: sphingomyelin;; AABA: α-aminobutyric acid
